# Bioinspired palladium-doped manganese oxide nanocorns: a remarkable antimicrobial agent targeting phyto/animal pathogens

**DOI:** 10.1038/s41598-023-40822-1

**Published:** 2023-08-28

**Authors:** Sagar Vikal, Yogendra K. Gautam, Ashwani Kumar, Ajay Kumar, Jyoti Singh, Dharmendra Pratap, Beer Pal Singh, Neetu Singh

**Affiliations:** 1 Smart Materials and Sensors Laboratory, Department of Physics, Chaudhary Charan Singh University, Meerut, Uttar Pradesh 250004 India; 2 Nanoscience Laboratory, Institute Instrumentation Centre, IIT Roorkee, Roorkee, 247667 India; 3 Department of Biotechnology, Mewar Institute of Management, Ghaziabad, Uttar Pradesh 201012 India; 4 Plant Molecular Virology Laboratory, Department of Genetics and Plant Breeding, Chaudhary Charan Singh University, Meerut, Uttar Pradesh 250004 India; 5 Department of Physics, Graphic Era (Deemed to Be University), Dehradun, Uttarakhand 248002 India

**Keywords:** Biological techniques, Biophysics, Cell biology, Materials science

## Abstract

Microbial pathogens are known for causing great environmental stress, owing to which emerging challenges like lack of eco-friendly remediation measures, development of drug-resistant and mutational microbial strains, etc., warrants novel and green routes as a stepping stone to serve such concerns sustainably. In the present study, palladium (Pd) doped manganese (II, III) oxide (Mn_3_O_4_) nanoparticles (NPs) were synthesized using an aqueous *Syzygium aromaticum* bud (ASAB) extract. Preliminary phytochemical analysis of ASAB extract indicates the presence of polyphenolics such as phenols, alkaloids, and flavonoids that can act as potential capping agents in NPs synthesis, which was later confirmed in FTIR analysis of pure and Pd-doped Mn_3_O_4_ NPs. XRD, Raman, and XPS analyses confirmed the Pd doping in Mn_3_O_4_ NPs. FESEM and HRTEM study reveals the mixed morphologies dominated by nanocorns appearance. Zeta potential investigation reveals high stability of the synthesized NPs in colloidal solutions. The developed Pd-doped Mn_3_O_4_ NPs were tested against two fungal phytopathogens, i.e., *Sclerotinia sclerotiorum* and *Colletotrichum gloeosporioides*, known for causing great economic losses in yield and quality of different plant species. The antifungal activity of synthesized Pd‐doped Mn_3_O_4_ NPs displayed a dose‐dependent response with a maximum of ~92%, and ~72% inhibition was recorded against *S. sclerotiorum* and *C. gloeosporioides*, respectively, at 1000 ppm concentration. However, *C. gloeosporioides* demonstrated higher sensitivity to Pd‐doped Mn_3_O_4_ NPs upto 500 ppm) treatment than *S. sclerotiorum*. The prepared NPs also showed significant antibacterial activity against *Enterococcus faecalis*. The Pd-doped Mn_3_O_4_ NPs were effective even at low treatment doses, i.e., 50–100 ppm, with the highest Zone of inhibition obtained at 1000 ppm concentration. Our findings provide a novel, eco-benign, and cost-effective approach for formulating a nanomaterial composition offering multifaceted utilities as an effective antimicrobial agent.

## Introduction

Nanobiotechnology integrates biotechnology and nanotechnology, dealing with applications of nanomaterials in biological sciences^1–4^. With the advent of nanobiotechnology, nanostructures' biological and physiochemical properties are tuned to serve the most relevant areas of human welfare, like medicine and agriculture^5^. Among different types, metallic nanoparticles (MNPs) are one of the widely exploited antimicrobial nanomaterials against phyto- and animal pathogens^6–12^. Researchers have recently witnessed a growing interest in synthesizing biocompatible metal-based NPs, utilizing green chemistry and bioinspired fabrication routes^13–21^.

Biological synthesis offers an eco-friendly and cost-effective method for the fabrication of NPs^22^ and is preferred over conventional methods^23^. Different biogenic sources for the synthesis of MNPs, like bacteria, plants, algae, fungi, yeasts, and actinomycetes, cause considerable modifications in the properties of their corresponding metals^24^. Among such sources, plant-based bioinspired fabrication of NPs is one of the preferred approaches^24,25^. The bioactive compounds in plant extracts can act as potential reducing and capping agents in synthesizing MNPs^24,25^. Green synthesis of MNPs like silver, copper, gold, iron, titanium, zinc, platinum, palladium, etc., has been extensively investigated^26–28^. However, limited investigations for green synthesis of manganese (Mn) NPs have been reported so far^24^, despite various applications in catalysis, biomedicine, electronics, electrochemistry, energy, optics, biosensors, food, pharmaceutical, cosmetics, textile industries, etc.^24,29^.

Metal oxides such as ZnO, CuO, TiO_2_ and MnO etc. have also a great potential to have excellent antimicrobial activity^19^. Chamaecostus cuspidatus extract is used to green synthesis CeO_2_ and ZnO nanoparticles (NPs) and effective antibacterial activities. The anticancer effects of CeO_2_ and ZnO nanoparticles were investigated in human breast cancer cell lines^21^. Similarly, Green synthesis is used to prepare Cerium oxide nanoparticles (CeO_2_ NPs) from Artabotrys hexapetalus leaf extracts. The prepared NPs exhibit excellent antibacterial activity against a variety of bacterial species. The anticancer potential of the compound was studied against the (MCF-7) human breast cancer cell line^22^. In addition to that, Zinc oxide nanoparticle (ZnO NPs) was prepared utilizing starch in a single step green synthesis and had highly porous, novel hollow multi-sphere in morphology. Because of their morphology and porosity, the synthesized ZnO NPs can be employed in a variety of drug delivery applications^19^. Mn has been reported as the transition element with the third highest abundance on earth followed by iron and titanium^30^. Among various 3d transition metal-oxides, Mn-oxides (MnO, MnO_2_, Mn_2_O_3_, Mn_3_O_4_, and Mn_5_O_8_) have obtained key attention owing to their compositional and structural diversity^24,31^. Mn-oxides NPs also possess structural adaptability with varying physicochemical qualities^32^. Mn-oxide NPs have excessive potential for sustainable-nanotechnology research and innovation^24,30^. Mn-oxides can have applications in optoelectronics, magnetic storage devices, imaging contrast agents, magnetic materials, drug delivery, catalysis, wastewater treatment, solar cells, etc.^24^.

*Sclerotinia sclerotiorum* is a necrotrophic phytopathogen that harbors a broad host range and causes stem rot disease in different crops including soybean, oilseed rape, sunflower, tomato, etc., resulting huge losses of agricultural produce^33^. On the other hand, *C. gloeosporioides* follows the hemibiotrophic infection mode and is known for causing anthracnose in fruits like papaya, mango, avocado, apple, guava, banana, papaya, cashews, grapes, pitaya, etc., resulting in serious postharvest losses^34,35^. Although chemical fungicides have been utilized for their control, their indiscriminate utilization has serious environmental consequences that necessitate the search for novel and eco-friendly alternatives^36^. *E. faecalis* is known to colonize the human intestine, and its occurrence in aquatic bodies implies fecal contamination^37^. These bacteria have been reported as multidrug-resistance microbial pathogens associated with hospital-acquired infections^37,38^.

To combat such underlying issues, present study focused on developing nanoformulation comprised of Pd-doped Mn_3_O_4_ NPs through the green chemistry route as a potential tool to offer a broad spectrum of antimicrobial activity.

In the current study, green synthesis of Mn_3_O_4_ NPs was completed by using *Syzygium aromaticum* bud extract as a potential reducing and capping agent. Adding novelty to this work, attempts were made to modify the structural attributes of Mn_3_O_4_ NPs through Pd doping to prioritize their multifaced applications in sectors such as agriculture, environment and medicine. To the best of our knowledge and available literature, this is the first report on the bioinspired fabrication of nanocorn-like Pd-doped Mn_3_O_4_ NPs using ASAB extract.

This present work investigated the antimicrobial potential of bioinspired Pd-doped Mn_3_O_4_ NPs against *S. sclerotiorum*, *C. gloeosporioides*, and *E. faecalis*.

## Experimental

### Materials and methods

All the chemicals used in the present study (such as manganese chloride tetrahydrate, palladium chloride, sodium hydroxide, dextrose, agar, hydrochloric acid, potassium hydroxide, sodium chloride, yeast extract, beef extract, and peptone) were of analytical grade and utilized without any further purifications. The solutions and reagents were prepared in double-distilled water (DDW).

### Collection of plant material

The evenly looking dried flower buds of *S. aromaticum* were obtained from the nearby local market of Meerut (Uttar Pradesh, India). The taxonomical evaluation was performed by Prof. Vijai Malik, Head, Department of Botany, CCS University, Meerut (UP) (letter reference no. Bot/PB/380). Plant material was procured as per applicable Institutional, International, and National guidelines. The specimens were deposited in the University Herbarium at the Department of Botany (Accession no. Bot. 26 V_2_L_4_).

### Preparation of *S. aromaticum* bud extract

The procured flower buds were thoroughly washed with distilled water to remove dirt particles and dried at 40 °C for 48 h. The dried flower buds were homogenized into a fine powdered form and stored in an air-tight container until used. To prepare the extract, powdered flower buds were macerated in DDW (1:10, w/v) at 60 °C for 2 h. After cooling at room temperature, the aqueous solution of phytoextract was filtered using Whatman filter paper number 1 at stored at 4 °C temperature until use.

### Green synthesis of pure and Pd-doped Mn_3_O_4_ nanoparticles

**Figure 1:**
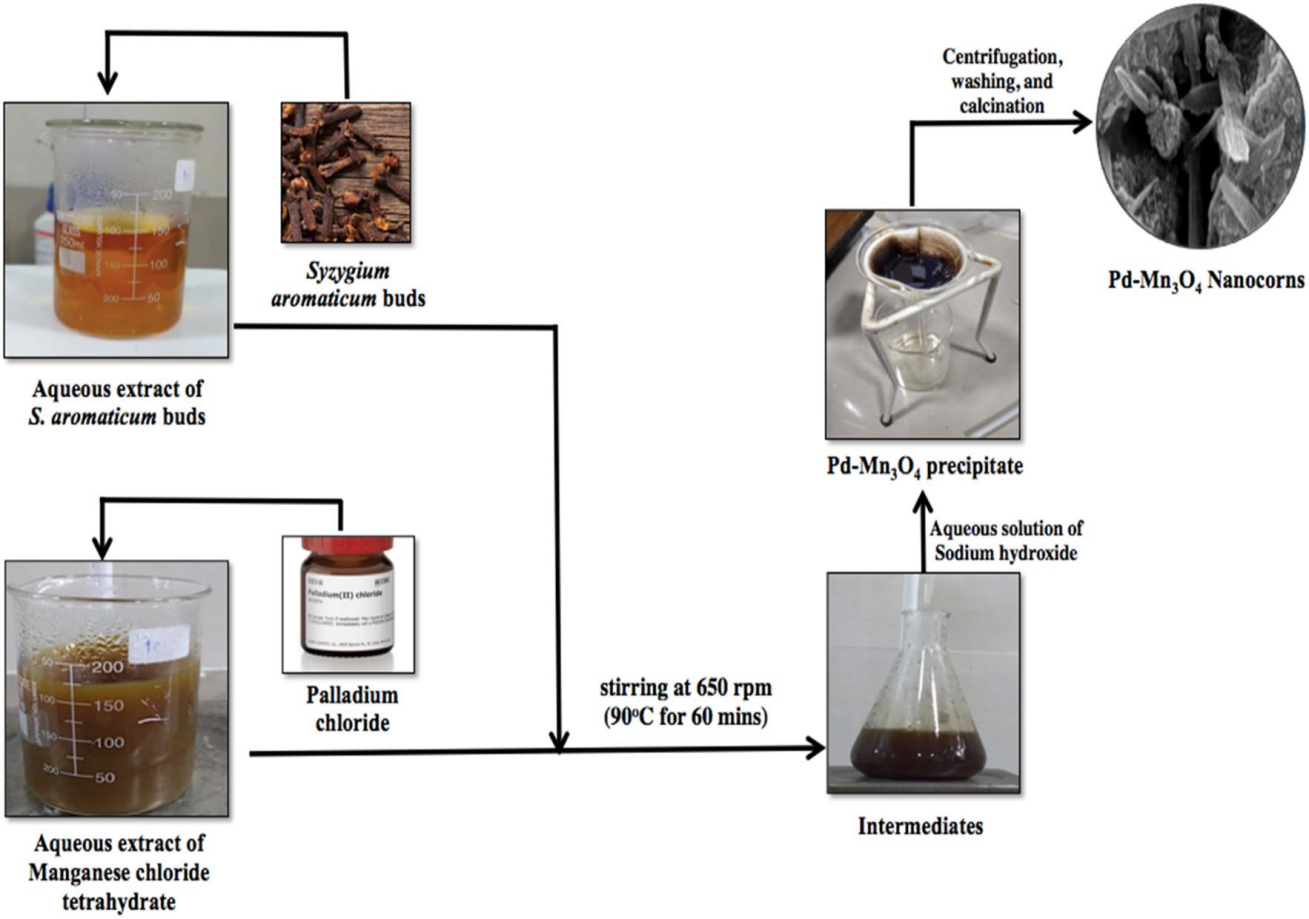
Schematic of the green synthesis of pure and Pd-doped Mn_3_O_4_ NPs.

The NPs were synthesized using a sol–gel method^39^, assisted by the addition of phytoextract as a potential source of capping agent (Fig. 1). Briefly, 2% (w/v) PdCl_2_ was added to the aqueous solution of MnCl_2_.4H_2_O (49 mM) followed by drop-wise addition of 10 ml aqueous *Syzygium aromaticum* bud extract under continuous stirring at 650 rpm at 90 °C for one hour. After getting the light brown color of the solution, 25 mM aqueous solution of NaOH was added drop-wise to adjust the pH. The alkali-mediated synthesis provides rapid precipitation leading to the mixture of manganese dihydroxide (Mn (OH)_2_) and manganese trihydroxide (Mn (OH)_3_)^40,41^. The appearance of a dark brownish-black precipitate indicated the formation of Pd-doped Mn_3_O_4_ NPs. The precipitate was collected using centrifugation at 5000 rpm for 10 min. The recovered precipitate gave multiple washings with ethanol and DDW to remove impurities, then dried at 150 °C for 3.5 h in a hot air oven. The finally dried residue was transformed into fine powder through mechanical grinding. The obtained Mn_3_O_4_ NPs were stored in an airtight bottle for further characterization. Except for the dopant addition, all other synthesis steps for pure Mn_3_O_4_ NPs were the same as those mentioned for Pd-doped Mn_3_O_4_ NPs. The key reaction steps in synthesizing Mn_3_O_4_ NPs are mentioned below^40^.

1 $${\text{MnCl}}_{{2}} \cdot {\text{4H}}_{{2}} {\text{O}} \rightleftharpoons {\text{Mn}}^{{{2} + }} + {\text{2Cl}}^{ - } + {\text{4H}}_{{2}} {\text{O}}$$

2 $${\text{2 Mn}}^{ + } \overset {{\text{NaOH}}} \rightleftharpoons {\text{Mn }}\left( {{\text{OH}}} \right)_{{2}} + {\text{ Mn }}\left( {{\text{OH}}} \right)_{{3}}$$

3 $${\text{Mn }}\left( {{\text{OH}}} \right)_{{2}} + {\text{ Mn}}\left( {{\text{OH}}} \right)_{{3}} \mathop{\longrightarrow}\limits^{{{\text{Calcination}}}}{\text{Mn}}_{{3}} {\text{O}}_{{4}} + {\text{4H}}_{{2}} {\text{O}}$$

### Characterization of synthesized nanoparticles

XRD analysis was performed to study the crystallinity and phases of prepared NPs (Bruker AXS, D8 Advance). The phase transitions and chemical composition of synthesized NPs were examined by Raman spectroscopy. The morphology and elemental composition were studied by FESEM (FEI, Quanta 200F), and EDAX, respectively. FTIR analysis conducted on ASAB extract and synthesized NPs to investigate the role of bioactive compounds in the development of NPs. The zeta potential and structural properties were determined using Zetasizer (Malvern Nano ZS) and HRTEM/SAED, respectively. XPS analysis was conducted to study the composition, and oxidation states of Pd-doped Mn_3_O_4_ NPs. The optical properties of prepared NPs were determined by UV–Visible spectroscopy.

### Antimicrobial activity of nanoparticles

The antifungal activity of NPs was tested against *S. sclerotiorum* and *C. gloeosporioides* using the poisoned food technique. The fungal cultures were procured from Indian Type Culture Collection (ITCC), Division of Plant Pathology, Indian Agriculture Research Institute, New Delhi, India. The potato dextrose agar (PDA) media was prepared for fungal growth with following composition: dextrose (2% w/v), potato starch (0.4% w/v), and agar (1.5% w/v). The pH of 5.6 ± 0.2 was adjusted using 0.1N KOH/0.1N HCl. The synthesized NPs were dispersed in PDA media to get the desired concentrations (up to 1000 ppm). The media containing NPs was poured into a Petri plate. At the next step, a ~ 8 mm piece of actively growing mycelia from 5 to 8 days old pure cultures of *S. sclerotiorum* and *C. gloeosporioides* were placed in the middle of each plate and incubated for five days at ~ 25 ± 2 °C and ~ 28 ± 2 °C temperature, respectively. The media plates without NPs treatment served as the negative control, and plates with 2 mg/ml of carbendazim + mancozeb were designated as the positive control. The % of growth inhibition was calculated using the below formula:

4 $$\frac{{\left( {{\text{Growth}}\;{\text{in}}\;{\text{Control}}\;{\text{plates}} - {\text{Growth}}\;{\text{in}}\;{\text{treatment}}\;{\text{plates}}} \right)}}{{{\text{Growth}}\;{\text{in}}\;{\text{Control}}\;{\text{plates}}}}{ } \times { }100$$

The antibacterial activity of NPs was determined using agar disc diffusion assay (ADDA). The pure culture of *E. faecalis* was inoculated to freshly prepared nutrient broth media (NBM) and maintained at 37 °C for ~ 18 h. The composition of NBM was Yeast extract (0.2% w/v), Beef extract (0.1% w/v), Peptone (0.5% w/v), NaCl (0.5% w/v), and pH ~ 7.4 ± 0.2. 2% (w/v) agar was added to the nutrient broth for preparing solid media plates (SPM). The pure culture of *E. faecalis* at the active log phase was uniformly spread on SPM. The sterile filter paper discs of about 6 mm diameter, each dipped in various concentrations of NPs (0 to 1000 ppm), were placed on SPM. The DDW-dipped discs were served as a negative control. The Petri plates were placed in an incubator overnight, and the ZOI was measured in millimetre. All the experiments on antimicrobial activity were performed in triplicates under aseptic conditions in a laminar airflow chamber. The nutrient media, glassware, and other utilities were autoclaved at 121 °C for 15 min at 15 psi pressure before use to maintain aseptic conditions throughout the assay.

### Statistical analysis

All of the experiments were completed in triplicates and recorded data presented as mean ± standard deviation using Microsoft Excel®.

## Results and discussion

### Green synthesis of nanoparticles and FTIR analysis

**Figure 2:**
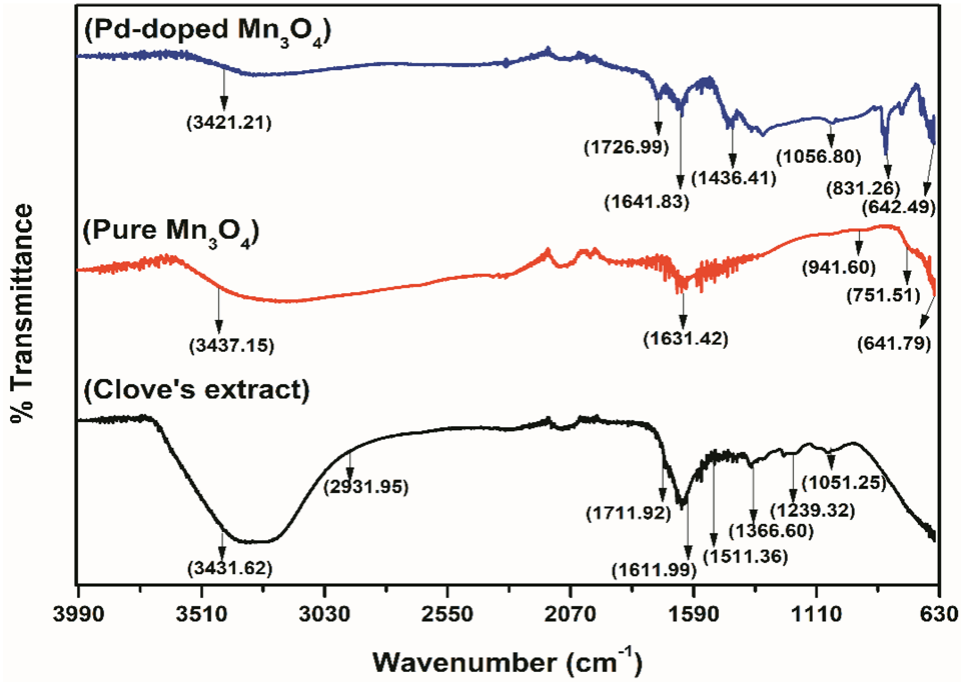
FTIR spectra of ASAB (clove) extract, pure, and Pd-doped Mn_3_O_4_ NPs.

Pure and Pd-doped Mn_3_O_4_ NPs were synthesized using an aqueous *Syzygium aromaticum* bud (ASAB) extract. Preliminary phytochemical analysis indicated the presence of phenols (ferric chloride test)^42^, flavonoids (lead acetate test)^43^, alkaloids (Wagner test), carbohydrates (fehling's test), and tannins (ferric chloride test)^44^. The results were in concordance with the findings of Jimoh et al.^43^, which established the suitability of the tested plant as a potential substrate for developing Phyto inspired nanoparticles^45–47^. Through FTIR analysis, Rajesh et al.^45^ predicted the role of metabolites present in *S. aromaticum* bud extract, such as flavonoids, tannins, alkaloids, and carotenoids, in the green synthesis of CuNPs. We have compared the FTIR spectra of ASAB extract, pure and Pd-doped Mn_3_O_4_ NPs to validate the capping and stabilizing potential of bioactive compounds present in phytoextract (Fig. 2).

FTIR spectrum of ASAB extract revealed a broad band at ~ 3431.62 cm^−1^, which corresponds to the OH group^45^, alkyl CH stretching (sp^3^), and C–O ester group was observed at ~ 2931.95 and ~ 1711.92 cm^−1^, respectively^48^. A sharp peak at ~ 1611.99 cm^−1^ belongs to –C = C aromatic stretching vibrations and C = O stretching vibrations of proteins denoting amide linkages ^45^. The aromatic groups were indicated at ~ 1511.36 cm^−1^^48^, while two separate peaks at ~ 1366.60 cm^−1^^45^ and ~ 1051.25 cm^−1^^48^ denoted the C–O group. These characteristic FTIR spectral peaks suggest for eugenol presence in ASAB extract^48^. The peak at ~ 640 cm^-1^ are attributed to the Mn–O in synthesized nanoparticles^49^. The peaks common to ASAB extract, pure and Pd-doped Mn_3_O_4_ NPs (~ 3450–3400, ~ 1750–1700, ~ 1650–1600, ~ 1400–1350, and ~ 1050 cm^-1^) depicted the capping potential of bioactive compounds in phytoextract.

### Structural analysis

**Figure 3:**
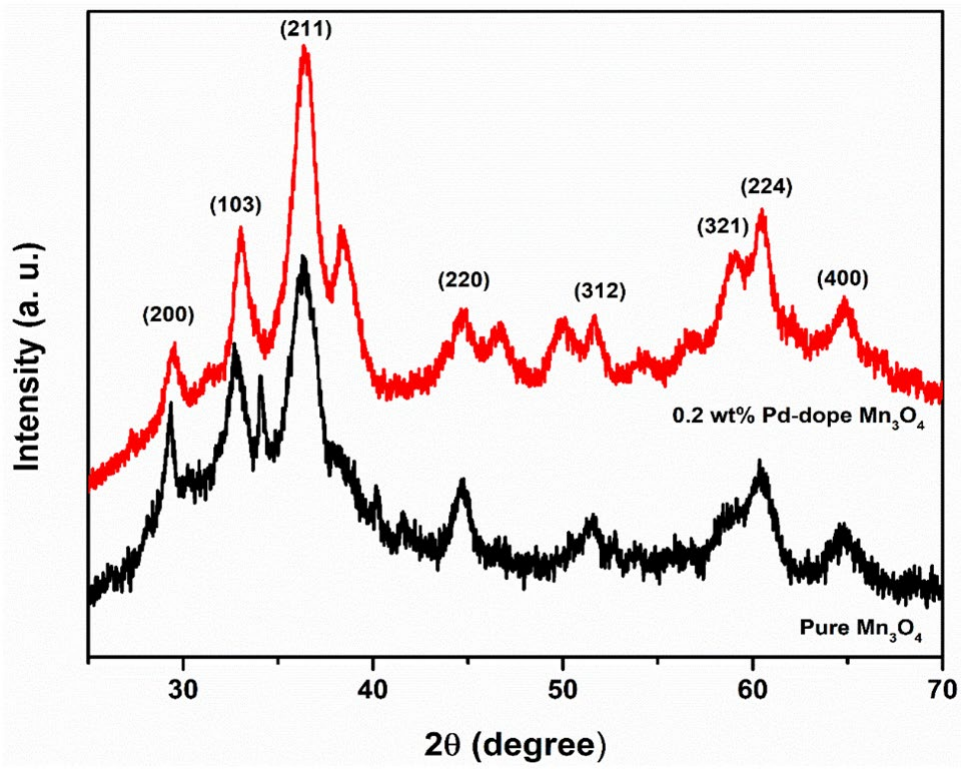
XRD spectra of pure and Pd-doped Mn_3_O_4_ NPs.

The XRD confirms the crystalline structure of the prepared manganese oxide NPs with two distinct phases, as shown in Fig. 3. The diffraction peaks of Mn_3_O_4_ samples at 2θ values of 31.04, 32.36, 36.13, 44.47, 53.83, 58.57, 59.90, and 64.69 matched well with the (200), (103), (211), (220), (312), (321), (224), and (400) crystal planes of Mn_3_O_4_ phase respectively^50^. The above (hkl) planes correspond to the Hausmannite phase of the Mn_3_O_4_ crystal structure (JCPDS 24–0734)^51^. The intensity of XRD peaks was decreased in the case of Pd doping, indicating peak shifting towards lower diffraction angles and crystalline lattice expansion^52^, suggesting the successful incorporation of Pd in Mn_3_O_4_. There is no shifting of XRD peaks in the case of Mn_3_O_4_ NPs, indicating that as-synthesized NPs are comprised of the tetragonal Hausmannite phase^53^. The sharp peaks confirmed the highly crystalline nature of NPs^54^. There were no other peaks in the XRD pattern that demonstrated the phase purity of the produced NPs^55^.

The crystallite size of synthesized NPs were calculated by Scherrer’s formula at a extremely intense peak (Eq. 5), and the values were ~ 32 and ~ 28 nm for pure and Pd-doped Mn_3_O_4_ NPs, respectively^56,57^. The Pd-doped Mn_3_O_4_ NPs were smaller than the pure Mn_3_O_4_ NPs, which could be owing to the fact that ionic radii of Pd (0.137 nm) are much larger than that of Mn (0.082 nm)^58^.

5 $$D = \frac{0.89\lambda }{{\beta cos\theta }}$$

where *D* is crystallite size, λ is the wavelength, θ is Bragg’s angle, and β is FWHM.

### Surface morphology and elemental analysis

**Figure 4:**
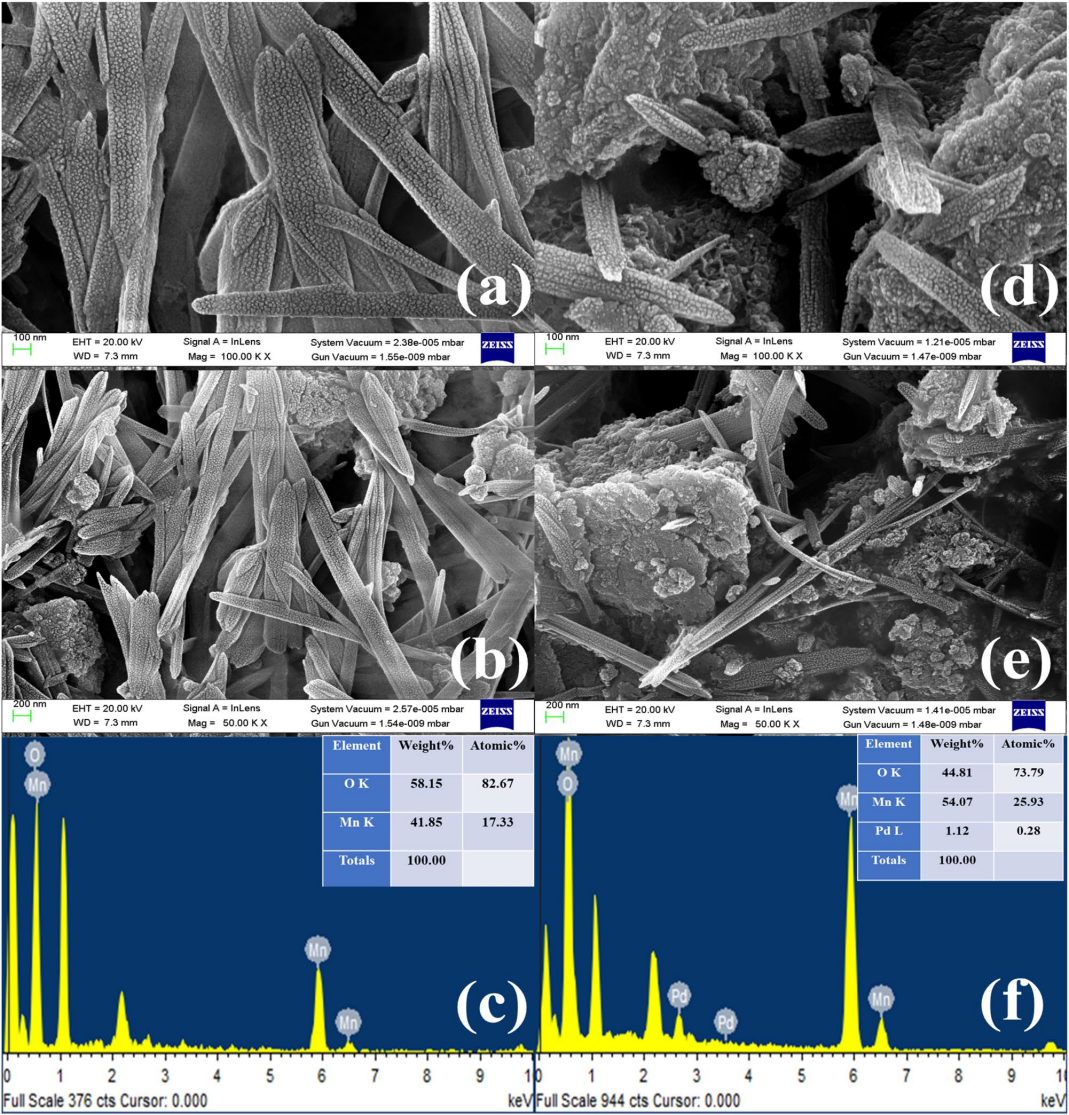
FESEM micrographs, and EDAX spectra of (**a**, **b, c**) pure Mn_3_O_4_ NPs and (**d, e, f**) Pd-doped Mn_3_O_4_ NPs.

The surface morphology of the pure Mn_3_O_4_ NPs in Fig. 4a, b appeared to be rod-like nanostructures. In contrast, Fig. 4d, e showed the surface of Pd doped-Mn_3_O_4_ NPs with the likely appearance of nanocorn-like structures. The morphological changes from rod (Mn_3_O_4_ NPs) to nanocorn-like nanocorn (Pd-doped Mn_3_O_4_ NPs) could be owing to the decoration of Mn_3_O_4_ with Pd^2+^. EDAX analysis revealed Pd, Mn, and O in their suitable stoichiometric proportion, as given in Fig. 4c, f ^59^.

### Optical studies

**Figure 5:**
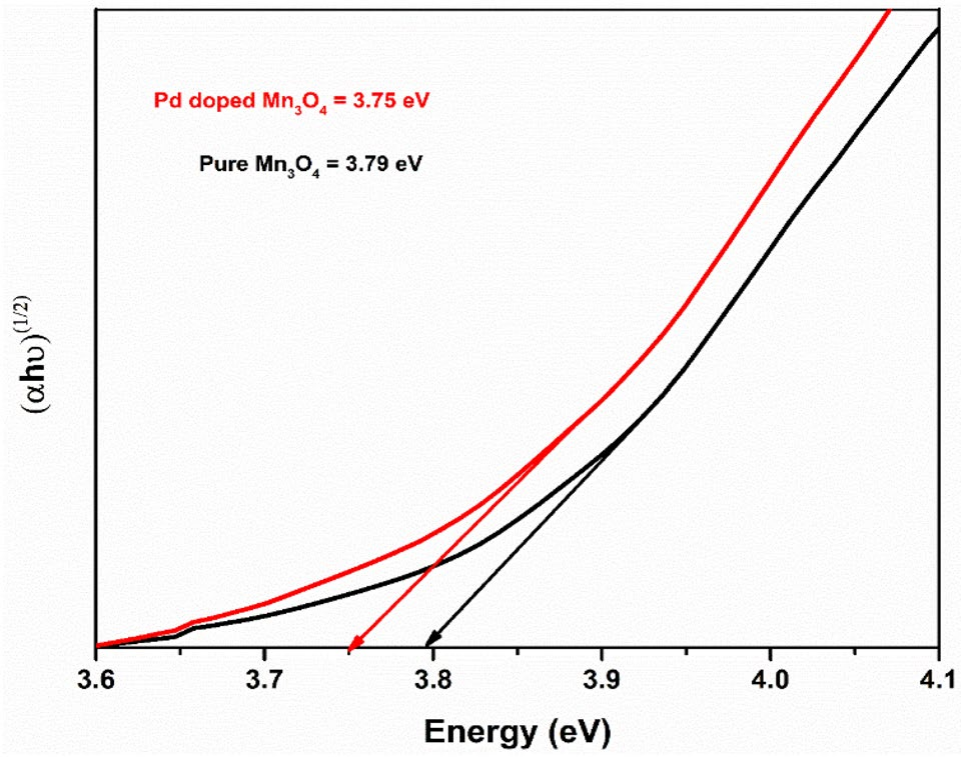
Tauc’s plot of pure and Pd-doped Mn_3_O_4_ NPs.

The absorption spectra of pure and Pd-doped Mn_3_O_4_ NPs were recorded at room temperature using a UV–visible spectrophotometer. In absorption spectra, the optical absorption edge was shifted to a higher wavelength region with Pd doping; consequently, the red shift was noticed in Pd-doped Mn_3_O_4_ NPs. The energy band gap of developed NPs was calculated by Tauc’s relation (Eq. 6)^60^.

6 $$\left( {\alpha {\text{h}}\nu } \right)^{{\text{n}}} = {\text{ B}}\left( {{\text{h}}\nu - {\text{E}}_{{\text{g}}} } \right),$$

where α, hν, E_g_, and B are the absorption coefficient, photon energy, the band gap energy, and constant, respectively. The value of index ‘n’ calculated from Tauc’s Plot was 2 (Fig. 5). The estimated band gap was ~ 3.79 and ~ 3.75 eV for pure and Pd-doped Mn_3_O_4_ NPs, respectively, which is in agreement with the previous reports for Mn_3_O_4_ NPs^61^. It was noticed that the band gap decreased in Pd-doped Mn_3_O_4_ NPs, compared to pure Mn_3_O_4_ NPs. A red shift was observed in the band gap of Pd-doped Mn_3_O_4_ NPs. This may be due to intermediate levels forming between the CB and VB of the host Mn_3_O_4_ matrix^62,63^. Pd atoms act as an accepter to decrease the band gap of Mn_3_O_4_ NPs^64,65^. Therefore, variation in the energy band gap of Mn_3_O_4_ NPs by Pd doping may have applications in photocatalytic activity^66^.

### Electronic states and elemental composition analysis

**Figure 6:**
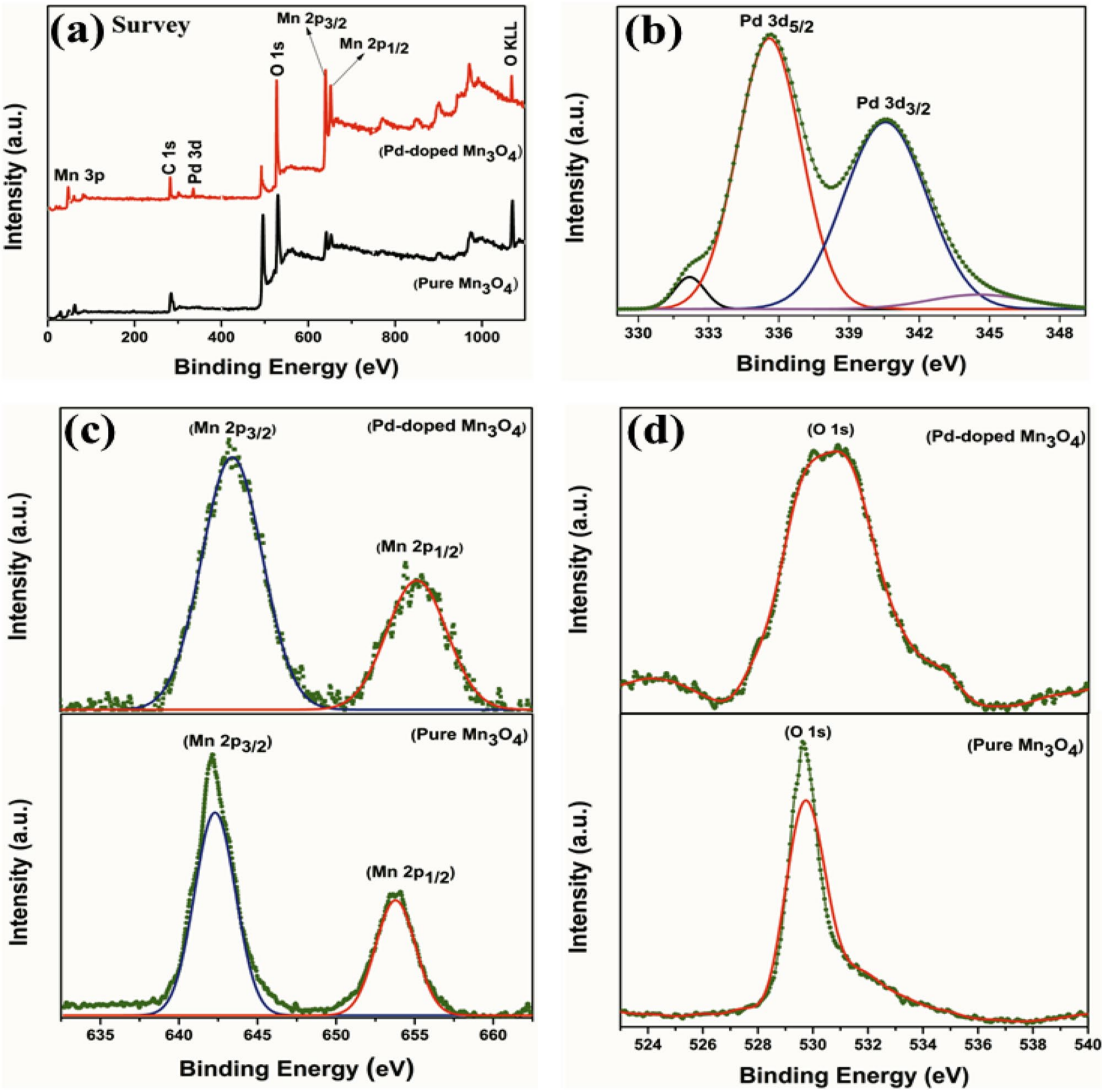
(**a**) The full XPS survey graph of pure and Pd–doped Mn_3_O_4_; (**b**) Pd 3d (**c**) Mn 2p and (**d**) O 1 s.

The oxidation states of Pd-doped Mn_3_O_4_ NPs were determined by XPS analysis^67^. The survey spectrum (Fig. 6a) revealed the presence of Pd, Mn, and O, confirming their existence in the product, i.e., Pd-doped Mn_3_O_4_ NPs. Further analysis of the Pd 3d spectrum showed a doublet feature^68^, providing evidence of Pd species' presence in the material. The peaks observed at 332.17 eV and 335.56 eV in the Pd 3d_5/2_ region, along with 340.57 eV and 344.58 eV in the Pd 3d_3/2_ region, corresponded to Pd (II) and Pd (IV) states^68^. Moving on to the Mn 2p core-level spectrum, two distinct peaks were observed at binding energies of 654.94 eV, 653.56 eV and 643.53 eV, 642.13 eV for pure and Pd-doped Mn_3_O_4_ nanocorns, respectively. These peaks were associated with Mn 2p_1/2_ and Mn 2p_3/2_ in Mn_3_O_4_ NPs and indicating a spin-orbital splitting of 11.4 eV (Fig. 6b)^69,70^. Additionally, the O1s spectrum peaked at 529.68 eV for pure Mn_3_O_4_ NPs and 532.89 eV for Pd-doped Mn_3_O_4_ NPs (Fig. 6d)^70^. This peak confirmed the presence of oxygen in both materials. The XPS analysis provided conclusive evidence that the prepared manganese oxide material was indeed Pd-doped Mn_3_O_4_ NPs, with the oxidation states of Pd (II) and Pd (IV) and specific Mn 2p states characteristic of Mn_3_O_4_.

### Transmission electron microscopy analysis

**Figure 7:**
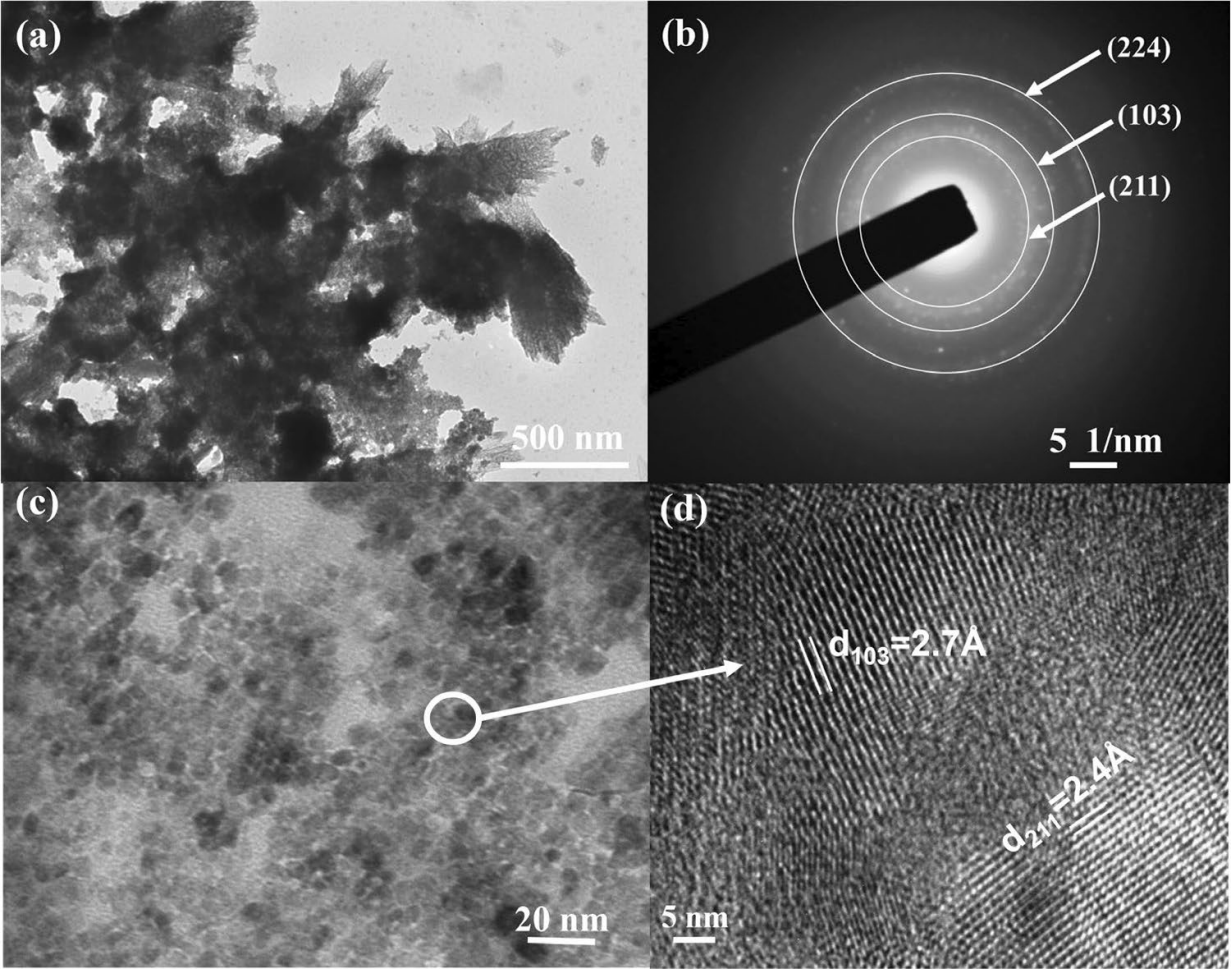
Electron micrographs showing Pd-doped Mn_3_O_4_ NPs,: (**a**) TEM image, (**b**) SAED pattern, (**c**) HRTEM image, and (**d**) interplanar lattice spacing.

The TEM micrograph of the green synthesized Mn_3_O_4_ NPs in Fig. 7a shows that the Mn_3_O_4_ NPs were composed of nearly uniform types of particles. The SAED patterns of Mn_3_O_4_ NPs in Fig. 7b displayed bright rings with some bright spots, suggesting the high crystallinity of the materials^71^. Figure 7c represents the high-resolution TEM images of the Pd-doped Mn_3_O_4_ NPs, and the magnified calibrated lattice fringes of Mn_3_O_4_ NPs for the crystal plane of (103) and (211) revealed an interplanar spacing (d-spacing) of 2.7 and 2.4 Å in Fig. 7d. These planes were also observed in the XRD analysis, and the reduction of the d-spacing of these planes is in good agreement with the shifting of the XRD peaks. Therefore, these findings indicate the successful formation of Pd-doped Mn_3_O_4_ NPs, which was also consistent with the XRD patterns^72,73^.

### Raman Spectroscopy analysis

**Figure 8:**
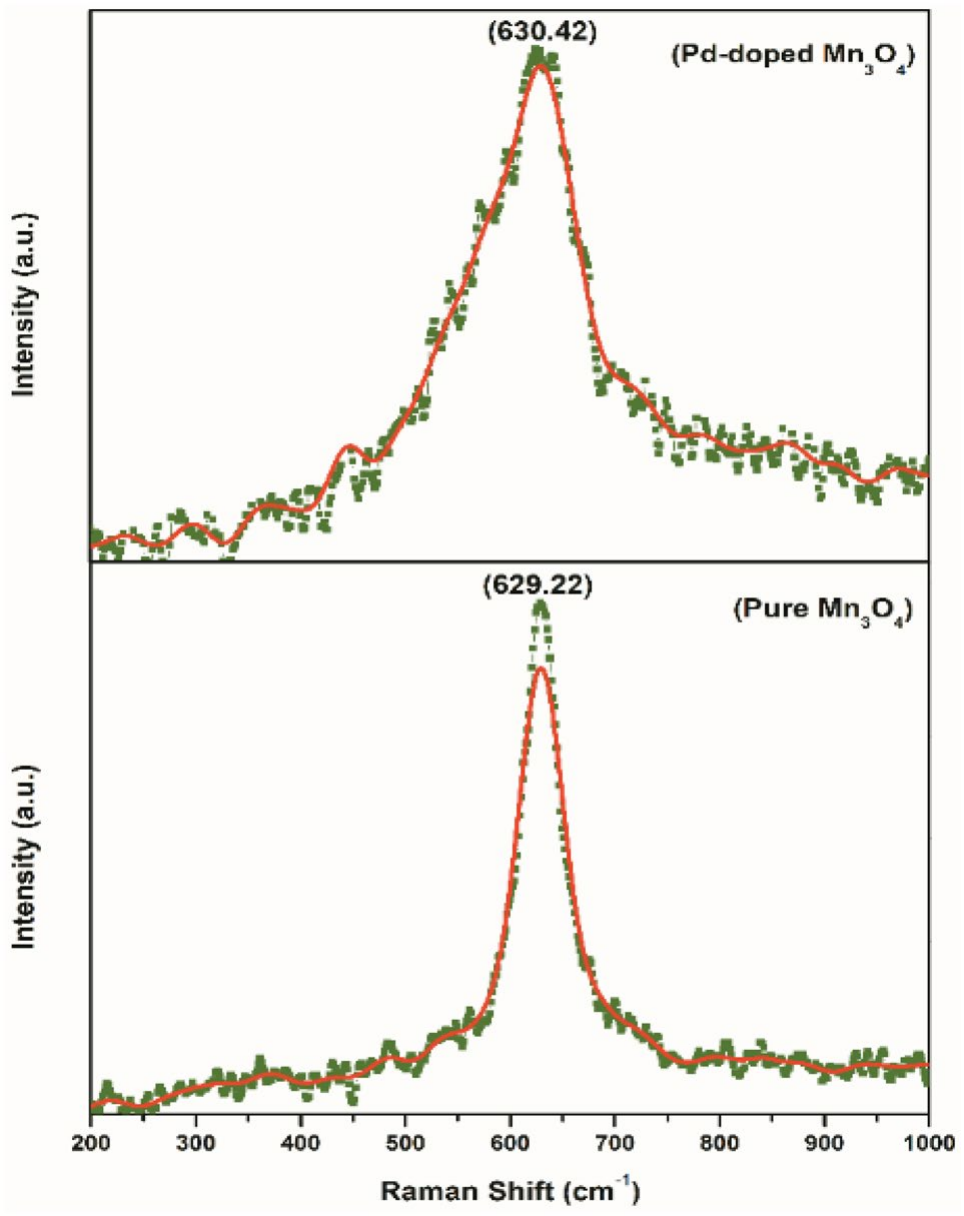
Raman spectra of pure and Pd-doped Mn_3_O_4_ nanoparticles.

Figure 8 presents the Raman spectra of pure and Pd-doped Mn_3_O_4_ NPs. Two characteristic peaks at ~ 629 and ~ 630 cm^−1^ were observed, corresponding to the skeletal vibrations for pure and Pd-doped samples, respectively. The strongest peaks at ~ 629 and ~ 630 cm^−1^ are consistent with the reported data^74^ for both materials. These sharp peaks can be assigned to the A1g mode, representing the Mn–O breathing vibration of divalent manganese ions in tetrahedral coordination. This mode is a characteristic feature of Hausmannite^75,76^. The comparison of the Raman spectra between pure and Pd-doped Mn_3_O_4_ NPs reveals similarities in the characteristic peaks, indicating that the introduction of Pd did not significantly alter the skeletal vibrations and Mn–O breathing vibrations in the tetrahedral coordination. The Raman spectra analysis of pure and Pd-doped Mn_3_O_4_ NPs confirmed the presence of specific vibrational modes and provides key insights into the structural properties of these materials. The similarities in the Raman spectra between pure and Pd-doped samples suggested that the Pd-doping did not cause significant changes in the observed vibrational features.

### Zeta potential studies

**Figure 9:**
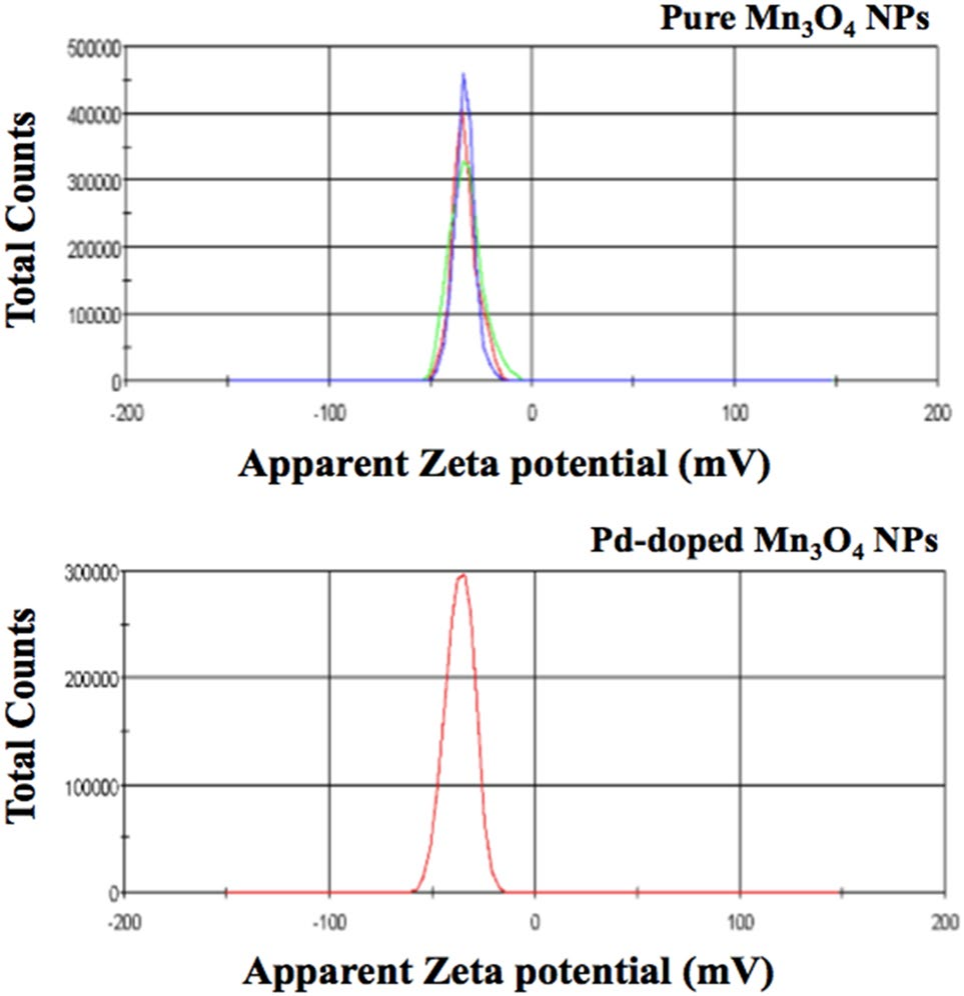
Zeta potential of pure and Pd-doped Mn_3_O_4_ NPs.

A particle's surface potential substantially impacts its dispersion stability^77^, which may also influence its bactericidal potential^78^. Without proper surfactants or capping agents, nanoparticles tend to agglomerate, and their surface area-to-volume ratio decreases due to their increased size^79^. The zeta potential studies allow us to investigate nanoparticles' surface charge and stability in colloidal solutions^80^ (Fig. 9). The surface charge of NPs can be influenced by the charged dopants^77^, which is also observed in the present study. We have obtained highly stable NPs (ZP > 30 mV)^81^, with recorded values of −33.2 ± 0.404 and −36.6 ± 1.74 mV for pure and Pd-doped Mn_3_O_4_, respectively. The NPs with greater ZP values (negative or positive) prevent agglomeration via electrostatic repulsion, hence conferring stability^80^.

## Antifungal activity

**Figure 10:**
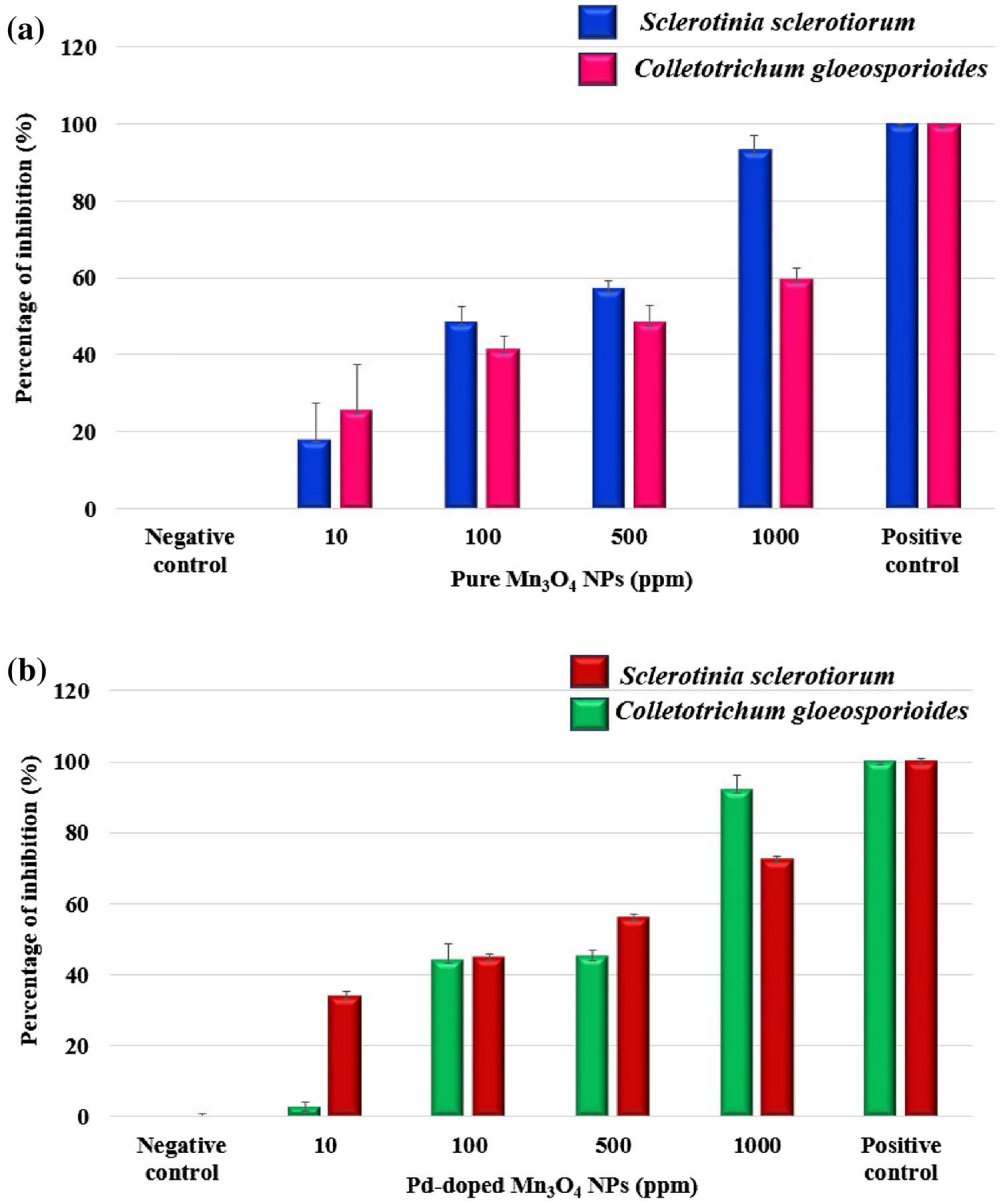
Effect of (**a**) pure Mn_3_O_4_ NPs, and (**b**) Pd- doped Mn_3_O_4_ NPs on growth of *S. sclerotiorum*, and *C. gloeosporioides.*

**Figure 11:**
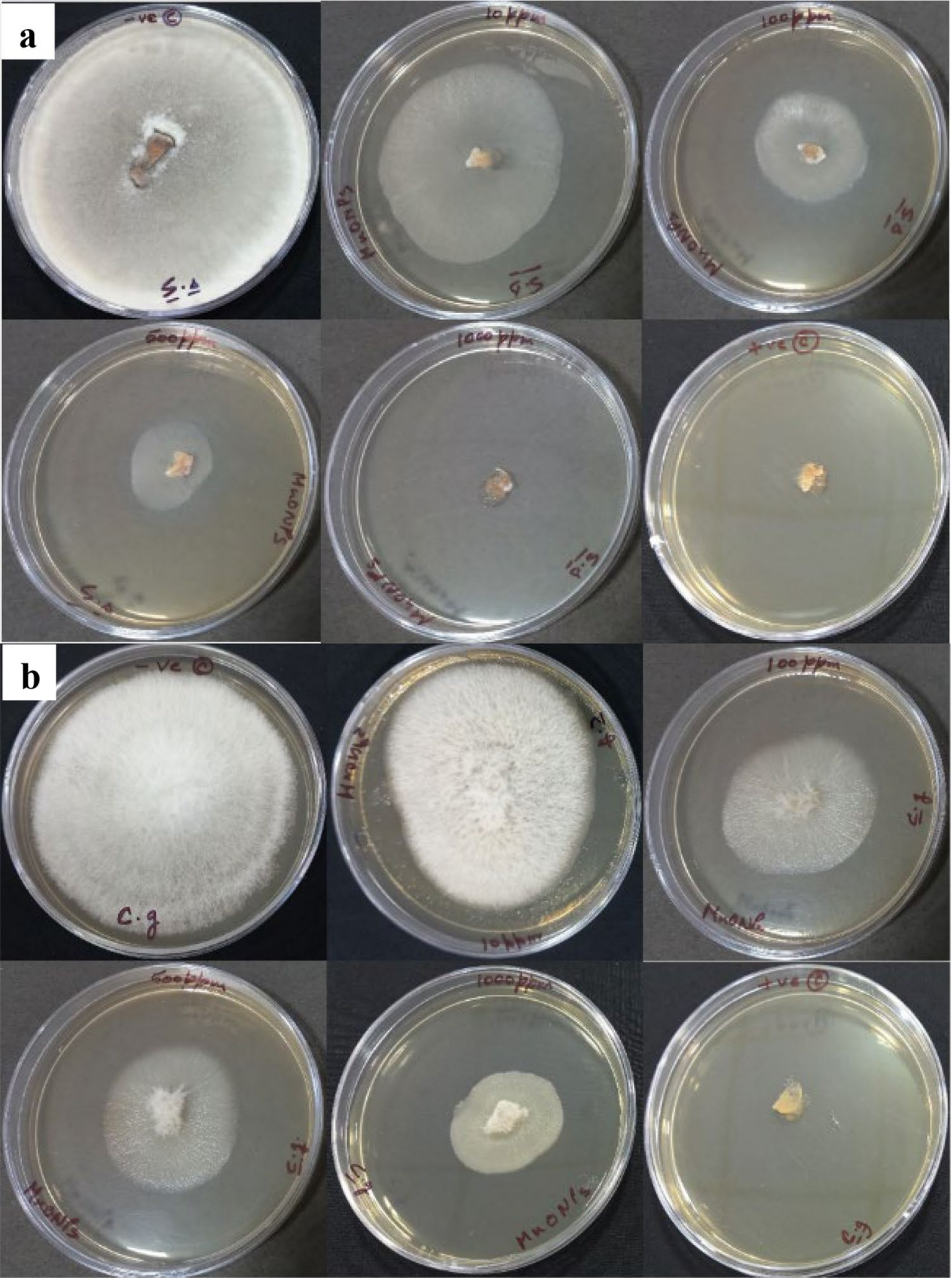
Antifungal activity of pure and Pd-doped Mn_3_O_4_ NPs against (**a**) *S. sclerotiorum*, and (**b**) *C. gloeosporioides.*

The developed NPs showed mycelium growth inhibition in a dose-dependent manner. In the case of Mn_3_O_4_, NPs, we have observed maximum antifungal activity at 1000-ppm concentration with over 50% inhibitions in the growth of S. sclerotiorum and C. gloeosporioides was recorded at 500 ppm dose (Fig. 10). Overall, S. sclerotiorum exhibited higher sensitivity to the Mn_3_O_4_ NPs treatment than C. gloeosporioides. The inhibition of mycelial growth in the case of Pd-doped Mn_3_O_4_ NPs was higher than pure Mn_3_O_4_ NPs against both fungal strains (Fig. 11). This could be due to the significant modification in structural properties of Mn_3_O_4_ NPs as a result of Pd doping, such as the reduction in crystallite size and nanocorn-like morphology. At 1000 ppm concentration, Pd-doped Mn_3_O_4_ NPs caused ~ 92% and ~ 72% growth inhibition of *S. sclerotiorum* and *C. gloeosporioides*, respectively. Interestingly, *C. gloeosporioides* was more sensitive to Pd-doped Mn_3_O_4_ NPs treatment, specifically at lower doses, and showed ~ 65%, ~ 23%, and ~ 10% higher inhibition compared to pure Mn_3_O_4_ NPs at 500, 100, and 10 ppm concentration, respectively. The Pd-doped Mn_3_O_4_ NPs at 1000 ppm showed antifungal activity comparable to those of positive control (2 mg/ml of carbendazim + mancozeb; commercial chemical grade fungicide formulation). Hence, doping Mn_3_O_4_ NPs with Pd favoured their antifungal potential^66^, which is highly explicitly recommended in the case of *C. gloeosporioides* to decrease the effective dose. Overall, the bioinspired fabrication of nanocorn-like Pd doped Mn_3_O_4_ NPs can be used as an effective antifungal nano-pesticide against different necrotrophic and hemibiotrophic phytopathogens, known for causing enormous loss to agricultural food crops globally.

## Antibacterial activity

**Figure 12:**
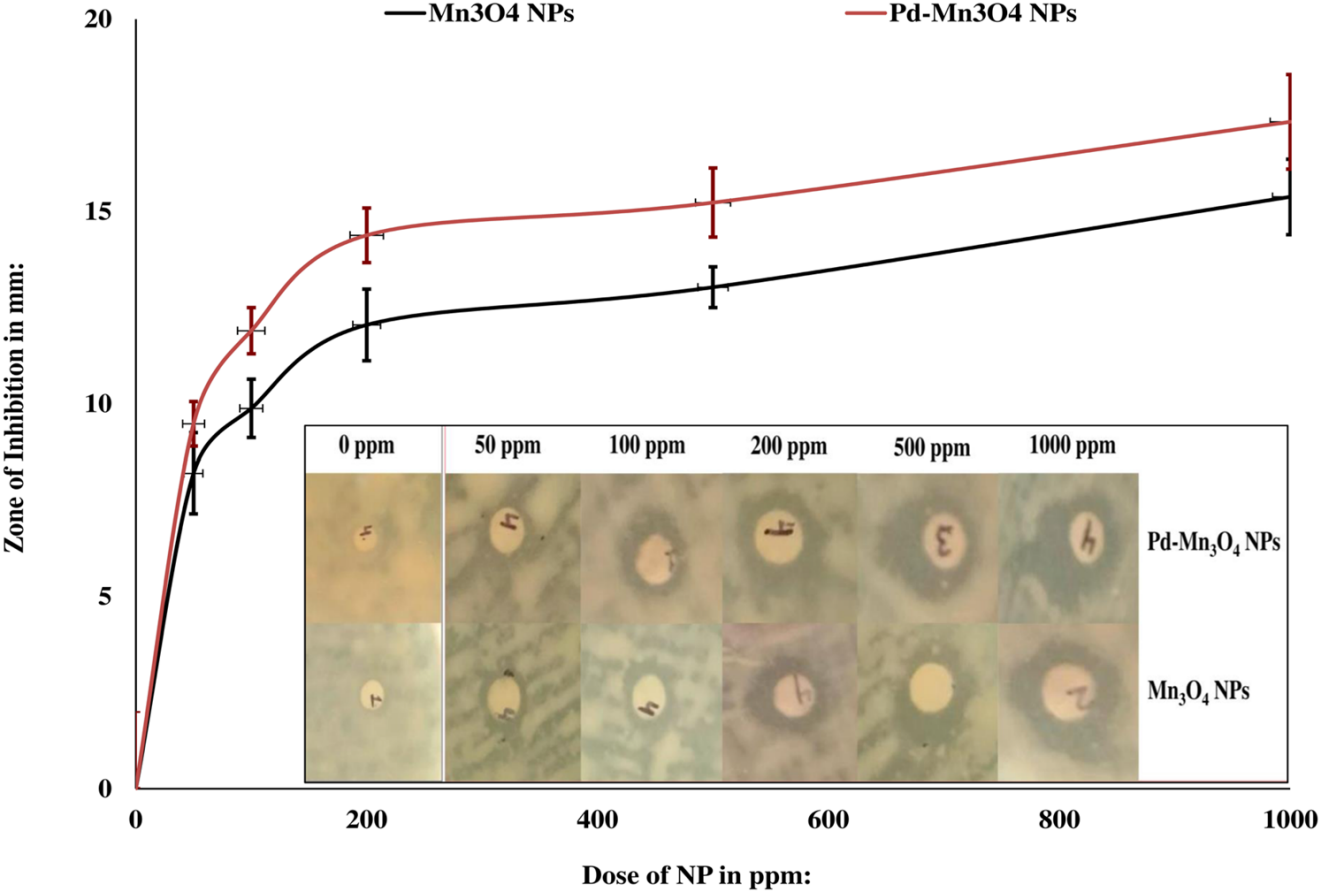
Antibacterial activity of pure and Pd-doped Mn_3_O_4_ NPs against *E. faecalis.*

The antibacterial activity of pure and Pd-doped Mn_3_O_4_ NPs was also investigated against *E. faecalis* to establish their broad spectrum of antimicrobial potential, in terms of pathogen diversity, i.e., phytopathogens and animal pathogens. As stated earlier, *E. faecalis* is a well-known human pathogen known for hospital acquired infections. This bacterium colonizes the intestine of animals including humans, and its presence in waterbodies is an indicative of fecal contamination. The resistance of *E. faecalis* against various antibiotics necessitated the search of novel materials possessing significant antibacterial potential against such nosocomial pathogens. In the present work, both pure and Pd-doped Mn_3_O_4_ NPs showed dose-dependent increment in ZOI (Fig. 12). The ZOI values showed effect of Pd doping on improving antibacterial activity of Mn_3_O_4_ NPs. When compared to pure Mn_3_O_4_ NPs, Pd doping showed ~ 14%, ~ 17%, and ~ 16% higher ZOI values at 50, 100, and 200 ppm doses of Mn_3_O_4_ NPs respectively.

## Mechanism of antimicrobial activity of Pd-doped Mn_3_O_4_ NPs

**Figure 13:**
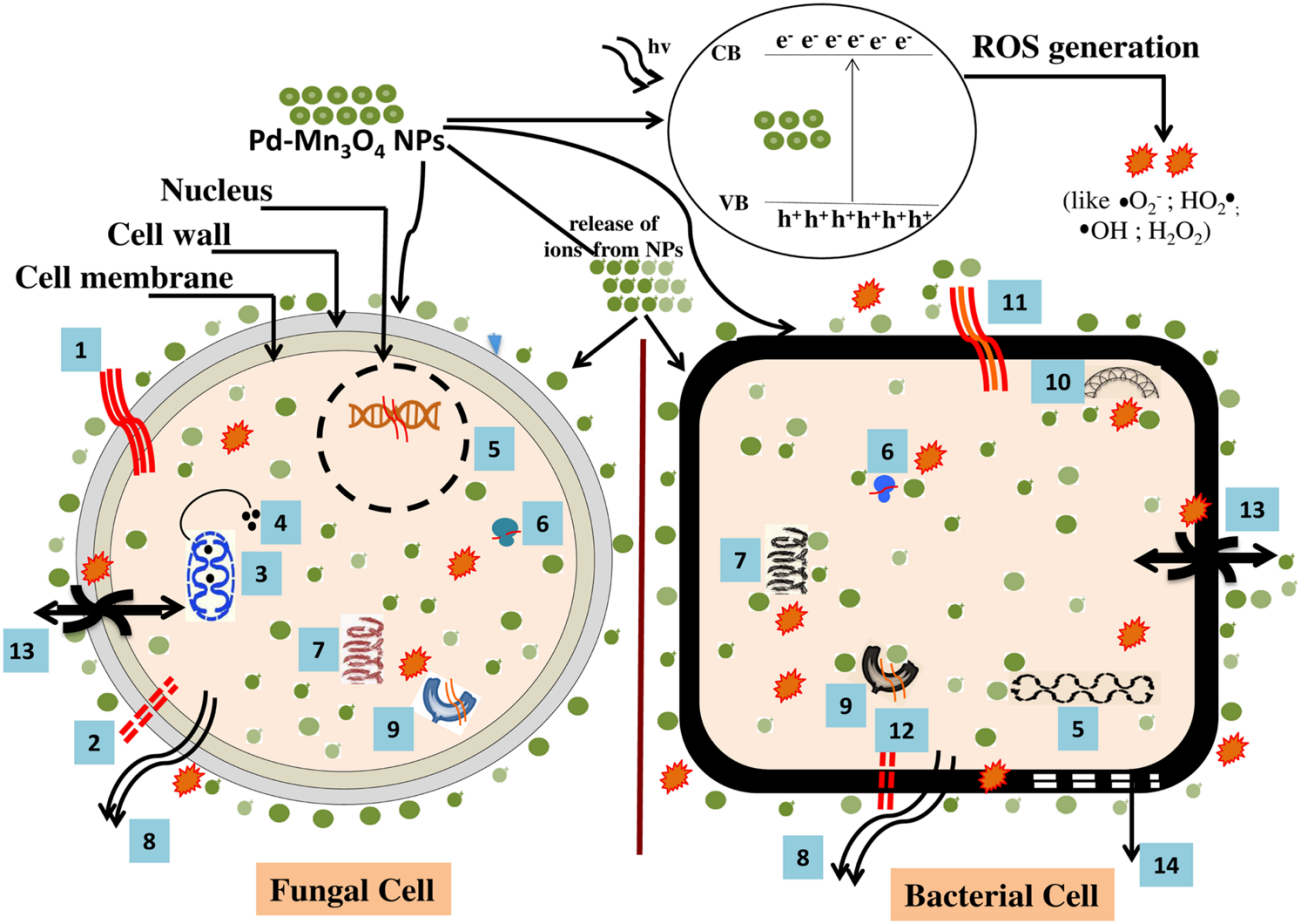
Schematic representation highligting plausible mechanisms involved in antimicrobial action of Pd-doped Mn_3_O_4_ NPs (1. breakdown of cell wall and cell membrane; 2. perforations in cell wall and cell membrane; 3. mitochondrial damage; 4. release of cytochrome-c from mitochondria to cytosol, increase levels of metacaspase and promotes cell death; 5. chromosomal DNA damage; 6. ribosome disassembly; 7. Protein damage; 8. release of cellular contents; 9. denaturation of enzymes; 10. plasmid DNA damage; 11. disruption of cell membrane; 12. perforation in cell membrane; 13. interference in ETC and damage to protein-efflux pump; 14. destruction of membranous proteins; CB = conduction band, VB = valance band; ROS: reactive oxygen species).

The plausible routes of inducing antimicrobial activity by Pd-doped Mn_3_O_4_ NPs are illustrated in Fig. 13^36,66,82^. The NPs in fungal cells usually gain entry via diffusion and endocytosis^83^ and may cause growth inhibition through multiple actions such as DNA damage, protein denaturation, breakdown of the cell wall and cell membrane, ROS-mediated lipid peroxidation, ribosome disassembly, denaturation of enzymes, perforations in the cell wall and cell membrane, mitochondrial damage, release of cytochrome-c from mitochondria to cytosol, and increase levels of metacaspase and promotes cell death^36^. Similar effects have been proposed in the case of bacterial cells where NPs can cause protein and enzyme denaturation, damage to chromosomal and plasmid DNA, ribosomal depolymerization, interference in ETC, the release of cellular contents, disruption of the cell membrane, etc.^84,85^.

**Figure 14:**
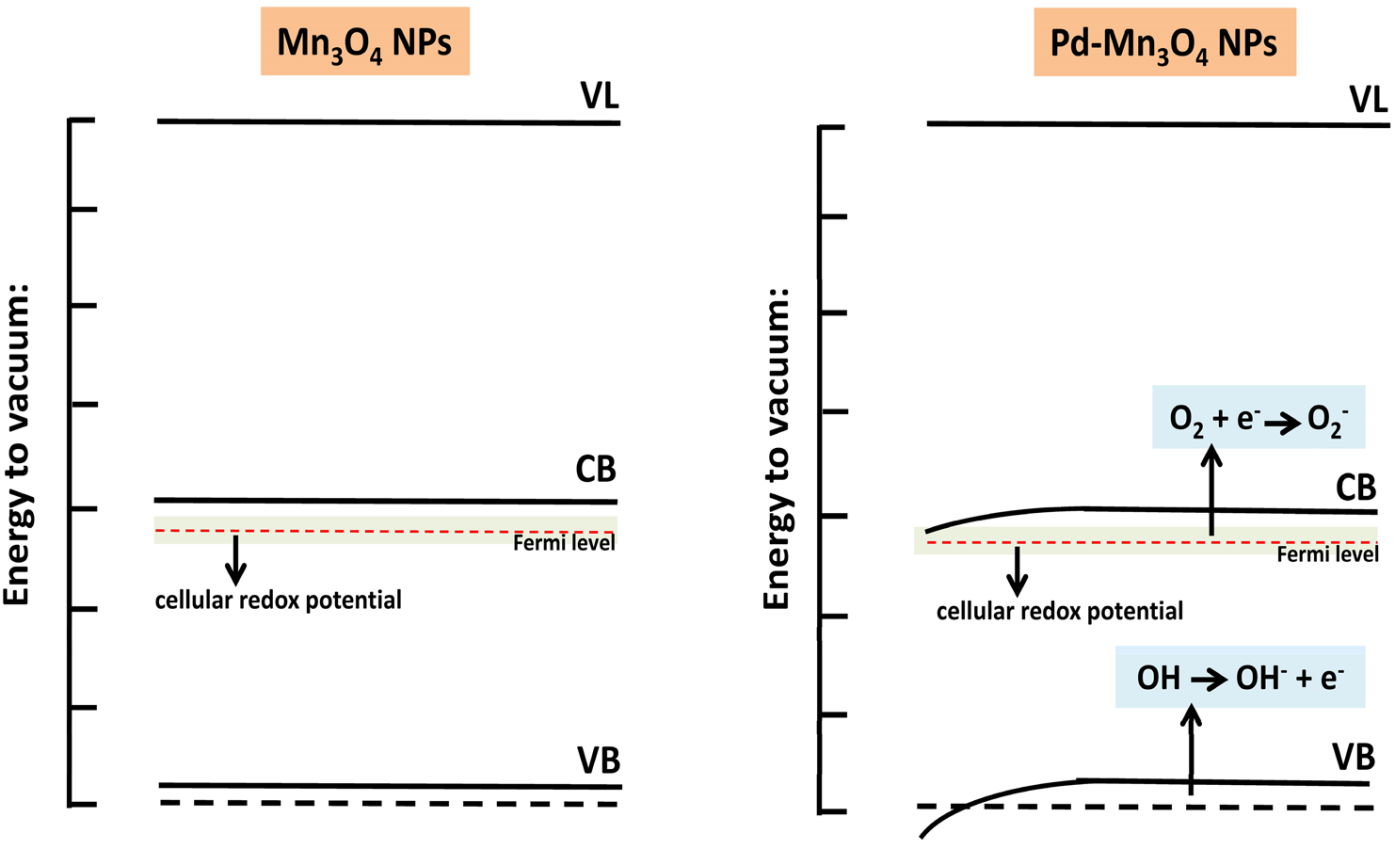
Effect of doping on Fermi levels leading to ROS generation (VL = vacuum level, CB = conduction band, VB = valance band) (conceptualized and redrawn from^39^).

In general, NPs have direct and indirect effects on microbial cells. The direct damage occurs via the electrostatic interaction of NPs with cell membrane resulting in membrane depolarization and loss of membrane integrity leading to the disruption in ETC and cell lysis^39,86^. The indirect damage to microbial cells is reported via ROS generation (Eqs. 7, 8, 9, 10, 11, 12)^39,87^. The doping in pure nanomaterial leads to lattice defects (alters band gap), causing overlapping of Fermi levels, variation in cellular redox potential, promotes ROS generation (Fig. 14) and can impart enhanced antimicrobial properties^39^, which was observed in the present study as well, where Pd-doped Mn_3_O_4_ NPs showed higher antifungal and antibacterial activity compared to pure NPs. In addition, doping can improve the binding capacity and cellular internalization ability of NPs. NPs generate ROS outside the cellular environment or can produce it inside the cell due to interference in ETC^39^. The oxygen molecules that are not reduced in the water get oxidized into free radicals (such as superoxide anion, singlet oxygen, or hydroxyl radicals) in mitochondria (Eqs. 7, 8, 9, 10, 11, 12)^39,88^. ROS causes alteration in protein structures, oxidative stress, lipid peroxidation, and DNA damage^88^.

7 $${\text{Pd-doped }} {\text{Mn}}_{{3}} {\text{O}}_{{4}} + {\text{ hv}} \to {\text{e}}^{ - } + {\text{h}}^{ + }$$

8 $${\text{h}}^{ + } {\text{H}}_{{2}} {\text{O}} \to {}^{ \bullet }{\text{OH}} + {\text{H}}^{ + }$$

9 $${\text{e}}^{ - } + {\text{ O}}_{{2}} \to {}^{ \bullet }{\text{O}}_{2}^{ - }$$

10 $${}^{ \bullet }{\text{O}}_{{2}}^{ - } + {\text{ H}}^{ + } \to {\text{ HO}}_{{2}}^{ \bullet }$$

11 $${\text{HO}}_{2}^{ \bullet } + {\text{ H}}^{ + } + {\text{ e}}^{ - } \to {\text{H}}_{{2}} {\text{O}}_{{2}}$$

12 $${\text{H}}_{{2}} {\text{O}}_{{2}} + {\text{e}}^{ - } \to {}^{ \bullet }{\text{OH }} + {\text{ HO}}^{ - }$$

## Conclusion

The present investigation demonstrates a successful green chemistry approach to synthesis pure and Pd-doped Mn_3_O_4_ NPs via utilizing an aqueous extract of *S. aromaticum* buds. Adding Pd in Mn_3_O_4_ resulted in significant changes to their structural attributes, including morphology, crystallite size, and lattice defects. The Pd-doped Mn_3_O_4_ NPs exhibited antimicrobial activity in a dose-dependent manner and provides higher inhibitory effects than pure Mn_3_O_4_ NPs against *S. sclerotiorum*, *C. gloeosporioides*, and *E. faecalis*. The outcome of this study provides a novel, cost-effective method to develop Pd-doped Mn_3_O_4_ based nanomaterials for highly effective antimicrobial applications against tested microbial pathogens. This breakthrough opens up new possibilities in the area of green nanotechnology to develop sustainable and multifaceted antimicrobial agents.

## Data Availability

All data generated or analyzed during this study are included within the article.

## Abbreviations

ADDA: Agar disc diffusion assay
ASAB: Aqueous *Syzygium aromaticum* bud
CB: Conduction band
DDW: Double-distilled water
DNA: Deoxy ribose nucleic acid
EDAX: Energy dispersive X-ray analysis
ETC: Electron transport chain
eV: Electron volt
FESEM: Field emission scanning electron microscopy
FTIR: Fourier-transform infrared spectroscopy
HCl: Hydrochloric acid
JCPDS: Joint Committee on Powder Diffraction Standards
KOH: Potassium hydroxide
mm: Milli meter
mM: Milli molar
Mn: Manganese
Mn_3_O_4_: Manganese (II, III) oxide
MnCl_2_·4H_2_O: Manganese chloride tetrahydrate
MNPs: Metallic nanoparticles
NaCl: Sodium chloride
NaOH: Sodium hydroxide
NBM: Nutrient broth media
nm: Nanometer
Pd: Palladium
PDA: Potato dextrose agar
PdCl_2_: Palladium chloride
ppm: Parts per million
ROS: Reactive oxygen species
SAED: Selected area electron diffraction
SPM: Solid media plates
TEM: Transmission electron microscopy
VB: Valence band
w/v: Weight by volume
XPS: X-ray Photoelectron Spectroscopy
XRD: X-Ray diffraction
ZOI: Zone of Inhibition
ZP: Zeta potential
